# New occurrence data of bryophytes and lichens from São Jorge Island (Azores, Macaronesia)

**DOI:** 10.3897/BDJ.14.e184350

**Published:** 2026-03-12

**Authors:** Gabriela M. Silveira, Rosalina Gabriel

**Affiliations:** 1 University of the Azores, Gaspar Frutuoso Foundation, Angra do Heroísmo, Azores, Portugal University of the Azores, Gaspar Frutuoso Foundation Angra do Heroísmo, Azores Portugal; 2 University of the Azores, CE3C- Centre for Ecology, Evolution and Environmental Changes/ Azorean Biodiversity Group | CHANGE – Global Change and Sustainability Institute, School of Agricultural and Environmental Sciences, Rua Capitão João d´Ávila, Pico da Urze, 9700-042, Angra do Heroísmo, Azores, Portugal University of the Azores, CE3C- Centre for Ecology, Evolution and Environmental Changes/ Azorean Biodiversity Group | CHANGE – Global Change and Sustainability Institute, School of Agricultural and Environmental Sciences Rua Capitão João d´Ávila, Pico da Urze, 9700-042, Angra do Heroísmo, Azores Portugal

**Keywords:** Azores, bryophytes, lichens, São Jorge Island, ad hoc collection, Macaronesia, conservation, protected area, species, substrates, IUCN, Wallacean shortfall

## Abstract

**Background:**

The Azorean Archipelago hosts an exceptionally rich cryptogamic flora, although several islands remain comparatively understudied. To address this gap, the present study presents records of bryophytes and lichens from São Jorge Island (SJO), based on opportunistic collections made during a MOVECLIM – Azores project expedition, from 29 July to 2 August 2014. Sampling was based on direct observation of noteworthy specimens and targeted areas with high substrate diversity and species richness. All collected material was subsequently examined and identified to species or subspecies level. Overall, the study aimed to: (i) document bryophyte and lichen species richness across major habitats; (ii) identify environmental drivers of species distribution; and (iii) increase the AZU Herbarium collection.

**New information:**

A total of 568 specimens were recorded, representing 111 species. Overall, the inventory comprises nine lichen species, three hornworts, 44 liverworts and 55 mosses; of these, 12 species are newly reported for SJO, including five lichens, one hornwort, one liverwort and five mosses. Three mosses are endemic to the Azores, six species are endemic to Macaronesia (four mosses; two liverworts) and twelve species are endemic to Europe (four mosses; eight liverworts). Most specimens were collected at elevations between 300 and 600 m and around 1000 m. Rocks and soil supported the greatest bryophyte diversity, whereas lichens were mostly collected on epiphytic substrates.

## Introduction

Oceanic island ecosystems, such as the Macaronesia Region, are unique places to study patterns of biodiversity, endemism and community formation (MacArthur and Wilson 1967, Gillespie et al. 2008, Borregaard et al. 2017), particularly amongst cryptogamic organisms. Bryophytes and lichens play key ecological roles in island ecosystems, contributing to water retention, nutrient cycling, soil formation, rock weathering, nitrogen input and the creation of microhabitats that support diverse invertebrate communities (Oksanen 2006, Ellis et al. 2021). As poikilohydric organisms, they respond quickly to fluctuations in humidity and temperature, making them especially sensitive to microclimatic variation and environmental disturbance (Gabriel et al. 2019, Coelho et al. 2023). This sensitivity explains their widespread use as bioindicators of habitat quality, climate change and anthropogenic disturbances (Rodrigues et al. 2024, Gabriel et al. 2024b).

In island systems, steep environmental gradients combined with long-term isolation often promote high species richness and turnover amongst cryptogamic groups (González-Mancebo et al. 2013). Although considerable efforts have been made to document cryptogamic diversity across Macaronesia (Gabriel et al. 2010, Rodrigues et al. 2024, ABP 2025), important knowledge gaps persist at the island scale. The Azores Archipelago, despite ranking amongst the three richest Atlantic archipelagos in bryophyte diversity (Mouton et al. 2023) and harbouring a substantial fraction of the Macaronesian bryophyte and lichen flora (Borges et al. 2010), remains unevenly surveyed. In particular, São Jorge stands out as one of the least studied islands, limiting our understanding of local biodiversity patterns and their broader biogeographic relevance.

## General description

### Purpose

The aim of this study is to enhance current knowledge of bryophyte and lichen flora of São Jorge Island (Azores) and to contribute new reference material to the Herbarium of the University of the Azores (AZU). All specimens were collected in 2014, using an opportunistic sampling approach, encompassing sites along the Island’s altitudinal gradient (30–1000 m a.s.l.) and a wide range of substrates.

## Project description

### Title

Improving the biodiversity knowledge in the Azores: new records of bryophytes and lichens for São Jorge Island

### Personnel

- Fieldwork (site selection and sample collection): Rosalina Gabriel (RG), with the help of Mário Boieiro, Débora S.G. Henriques, Fernando Pereira and Paulo A.V. Borges;
- Parataxonomists: Gabriela Melo da Silveira;
- Taxonomists: Rosalina Gabriel, António Félix Rodrigues;
- Voucher specimen management: Rosalina Gabriel, Gabriela Melo da Silveira;
- Database management: Rosalina Gabriel, Gabriela Melo da Silveira;
- Darwin Core databases management: Rosalina Gabriel.

### Study area description

The Azores Archipelago, consisting of nine islands, is located in the North Atlantic Ocean (36°55′–39°43′ N; 24°46′–31°16′ W), forming part of the Macaronesia biogeographic region, together with Madeira, the Canary Islands and Cape Verde. The Archipelago is strongly influenced by the Azores High, resulting in a temperate oceanic climate (Azevedo 2001), characterised by high annual precipitation, mild temperatures and consistently high relative humidity (Forjaz et al. 2004, DRAAC 2023).

Since human settlement, extensive land-use change, habitat degradation and the spread of exotic species have led to a strong reduction of native vegetation, particularly the Laurisilva Forest, which now covers approximately 10% of the Archipelago and persists mainly at higher elevations and in less accessible areas (Elias et al. 2016, Gabriel et al. 2024a). Despite this, the Archipelago’s isolation and environmental heterogeneity have promoted a rich endemic biota (Borges et al. 2009).

São Jorge Island (38°37′40″N, 28°01′02″W), located in the central group of the Archipelago, is a long and narrow island, dominated by a central mountain ridge reaching 1,053 m at Pico da Esperança. The island is characterised by steep coastal cliffs and low-lying fajãs, creating pronounced altitudinal and environmental gradients over short distances. Much of the Island’s remaining natural vegetation is included within the São Jorge Natural Park, established in 2011, which supports a high proportion of the Azorean endemic terrestrial taxa (GRA 2020).

**Figure 1.**
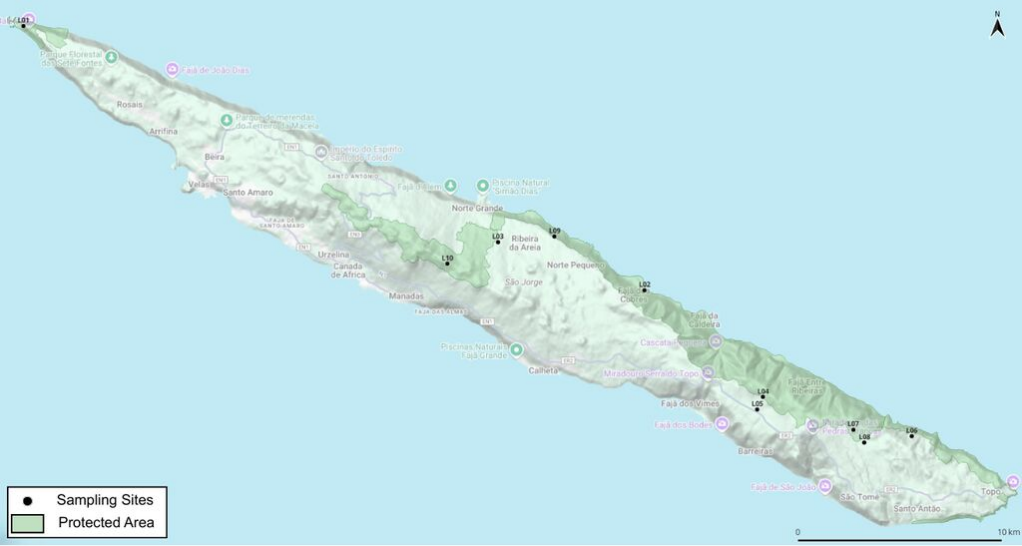
Map of São Jorge Island (Azores) showing the distribution of the 10 cryptogamic sampling sites in relation to the Island´s Natural Park (light green). Pico da Esperança, the island’s highest point, is located near site L10.

Surveyed locations are listed in Table 1, including site names, elevation, geographic coordinates and protection status, while the 10 main sampling sites are shown in Fig. 1.

### Design description

The sampling was conducted opportunistically, within the MOVECLIM project, between 29 July and 2 August 2014 on São Jorge Island. Fieldwork covered selected areas of the island's Natural Park and spanned a wide elevational range (30-1,000 m), including a variety of substrates (rupicolous, terricolous, humicolous, epiphytic, epixylic).

### Funding

This study was funded by ERANET BIOME MOVECLIM – Montane vegetation as listening posts for climate change (Regional Government of the Azores (GRA); grant M2.1.2/F/04/2011/NET). Additional support was provided by the GRA through PROJETO ESTAGIAR-L (G.M.S., grant PL2547575) and by Fundação para a Ciência e Tecnologia (FCT) through national and European funds by UID/00329/2025 Centre for Ecology, Evolution and Environmental Changes (CE3C).

## Sampling methods

### Study extent

At each locality, a qualitative survey of cryptogams was performed using direct visual inspection. The observer systematically examined accessible microhabitats and substrates. Representative specimens were collected and stored in paper envelopes with full field metadata. Laboratory identification followed standard morphological and anatomical protocols using dissection and compound microscopes.

### Sampling description

Sampling followed a non-systematic, opportunistic approach informed by the researcher’s prior knowledge and direct field observations (Broughton and Pocock 2022). Sampling points were selected to maximise microhabitat diversity, including live tree trunks, dead wood, exposed rocks and moist soils. Thus, it was possible to complement systematic approaches, increasing the number and quality of samples gathered.

Specimens were carefully collected using a knife or tweezers and placed in paper bags labelled with a unique code. When possible, each record was accompanied by in situ photographs and environmental metadata, including substrate type, slope, exposure, estimated light and humidity levels and surface roughness, scored using a scale inspired by Ellenberg indicator values (Gabriel and Bates 2005, Pakeman et al. 2019). Geographic coordinates and elevation values were also documented. Samples were dried at room temperature and stored under controlled conditions until analysis.

Taxonomic identification followed standard morphological and anatomical procedures using dissection, compound and stereoscopic microscopes, supported by taxonomic keys and floras (Paton 1999, Smith 2004, Schumacker and Váňa 2005, Casas et al. 2009, Atherton et al. 2010, Guerra et al. 2018, Casas et al. 2020). All data were recorded in a structured database following Darwin Core standards, including information on cover, sociability, reproductive structures and other traits.

### Quality control

All specimens were identified or revised by a taxonomical expert. The most challenging species were verified by specialists.

## Geographic coverage

### Description

The study was conducted on São Jorge Island (Azores, Portugal). The ten sampling sites were spread across the two municipalities of the island: Calheta and Velas.

### Coordinates

38.53 and 38.77 Latitude; -27.732 and -28.323 Longitude.

## Taxonomic coverage

### Description

Bryophytes (*Anthocerotophyta*, *Bryophyta* and *Marchantiophyta*) and lichens (*Ascomycota*).

## Temporal coverage

**Data range:** 2014-7-29 – 2014-8-02.

## Collection data

### Collection name

MOVECLIM-AZO-SJO_2014_Bryophytes and lichens from São Jorge Island - ad hoc

### Collection identifier

76349556-a70a-4ecc-88a7-cd085b6c875d | 05075c4b-7a65-4ddf-ad40-53dcfedb9343

### Parent collection identifier

AZU_Section Bryophytes | AZU_Section Lichens

### Specimen preservation method

Herbarium preservation

### Curatorial unit

Herbarium packet

## Usage licence

### Usage licence

Creative Commons Public Domain Waiver (CC-Zero)

## Data resources

### Data package title

The MOVECLIM – AZORES project: Ad hoc bryophyte and lichen from São Jorge Island (2014)

### Resource link

https://doi.org/10.15468/fhg8h5

### Alternative identifiers

https://ipt.gbif.pt/ipt/resource?r=crypto-azo_sjo_rg2014_adhoc

### Number of data sets

2

### Data set 1.

#### Data set name

event_table_v2

#### Data format

Darwin Core Archive

#### Character set

text unicode

#### Download URL

https://ipt.gbif.pt/ipt/resource?r=crypto-azo_sjo_rg2014_adhoc

#### Data format version

1.2

#### Description

The dataset was published through the Global Biodiversity Information Facility (GBIF) as a sampling-event dataset in Darwin Core Archive (DwCA) format (Gabriel and Silveira 2025), using the GBIF Integrated Publishing Toolkit (IPT v.2.5.6). The event core file comprises 120 sampling events (eventID) and is available for download, together with its metadata, via the Portuguese GBIF Portal.

**Data set 1.** 

Column label	Column description
eventID	A unique identifier for each sampling event, specific for the dataset.
parentEventID	Identifier of the parent sampling event to which this ad-hoc event belongs.
type	The nature or genre of the record, following the Darwin Core standard (e.g. Event).
datasetName	The name of the dataset from which the Event was derived. Current dataset: “MOVECLIM-AZO-SJO_2014_Bryophytes and lichens from São Jorge Island - ad hoc “.
samplingProtocol	Protocol used to collect the samples of the Event.
eventDate	The date or date range during which the Event occurred.
startDayOfYear	The calendar day (as an integer from 1 to 366) corresponding to the start of the Event.
day	The day of the month on which the Event occurred.
month	The month (as an integer) in which the Event occurred.
year	Year of which the samples were collected (2014).
habitat	Description of the habitat where the Event occurred.
continent	Name of the continent where the Location occurs.
country	Name of the country or major administrative unit where the Location occurs.
countryCode	The standard code for the country.
islandGroup	Name of the island group where the Event occurs.
island	Name of the island where the Event has occurred.
municipality	The full, unabbreviated name of the municipality or equivalent local administrative division where the Location occurs.
locality	The specific description of the place where the Event occurs.
verbatimElevation	The original description of the elevation (altitude, usually above sea level) of the Location.
decimalLatitude	The geographic latitude (in decimal degrees, positive for the Northern Hemisphere) of the geographic centre of a Location.
decimalLongitude	The geographic longitude (in decimal degrees, negative for the Western Hemisphere) of the geographic centre of a Location.
geodeticDatum	The geodetic reference system used for the coordinates.
coordinateUncertaintyInMetres	The horizontal uncertainty of the coordinates, expressed in metres.
coordinatePrecision	The precision of the latitude and longitude values, expressed in decimal degrees to five decimal places.
georeferenceSources	Source used to determine the geographic coordinates (GPS).

### Data set 2.

#### Data set name

occurrence_table_v2

#### Data format

Darwin Core Archive

#### Character set

text unicode

#### Download URL

https://ipt.gbif.pt/ipt/resource?r=crypto-azo_sjo_rg2014_adhoc

#### Data format version

1.2

#### Description

The dataset was published through the Global Biodiversity Information Facility (GBIF) as an occurrence dataset in Darwin Core Archive (DwCA) format (Gabriel and Silveira 2025), using the GBIF Integrated Publishing Toolkit (IPT v.2.5.6). The occurrence core file comprises 568 records (occurrenceID), mostly identified to species level, with six records identified to genus level and is available for download, together with its metadata, via the Portuguese GBIF Portal.

**Data set 2.** 

Column label	Column description
eventID	A unique identifier for each sampling event, specific for the dataset.
license	Reference to the legal document that defines how the resource can be used (Creative Commons Attribution (CC BY 4.0)).
institutionID	A globally unique identifier for the institution that has custody of the published data.
institutionCode	The acronym or code identifying the institution that holds or published the data (UAc).
collectionID	An identifier for the collection or dataset from which the record was derived.
collectionCode	The acronym or code identifying the collection from which the record was derived.
datasetName	The name of the dataset from which the record was derived. Current dataset: “MOVECLIM-AZO-SJO_2014_Bryophytes and lichens from São Jorge Island - ad hoc".
type	The nature or genre of the record, following the Darwin Core standard.
basisOfRecord	The specific nature of the record, using Darwin Core controlled vocabulary.
dynamicProperties	A list of additional attributes or observations related to the record, including colonisation status and IUCN conservation categories.
occurrenceID	A globally unique identifier for the Occurrence.
recordNumber	The identifier assigned to the Occurrence at the time of collection (commonly the collector’s field number).
recordedBy	The name(s) of the person(s), group(s) or organisation(s) who recorded or collected the Occurrence.
identifiedBy	The name(s) of the person(s) who identified the specimen(s).
dateIdentified	The date on which the specimen or taxon was identified.
disposition	The current status of the specimen in relation to its collection (in collection).
taxonRank	The taxonomic rank of the most specific name in the scientificName.
kingdom	The kingdom to which the taxon belongs.
phylum	The phylum (or division for plants) to which the taxon belongs.
class	The class to which the taxon belongs.
order	The order to which the taxon belongs.
family	The family to which the taxon belongs.
genus	The genus to which the taxon belongs.
specificEpithet	The species epithet (the second part of the scientific name).
infraspecificEpithet	The infraspecific epithet (e.g. subspecies or variety).
scientificNameAuthorship	The authorship information associated with the scientific name, formatted according to the relevant nomenclatural code.
scientificName	The full scientific name, including authorship and date information, if known.
organismQuantity	A numeric value representing the quantity or estimated cover of the organism(s) recorded.
organismQuantityType	The method or unit used to measure the organism quantity (Braun-Blanquet scale).
establishmentMeans	The process by which the organism became established at the location, using a controlled vocabulary (“native”, “endemic”).
occurrenceRemarks	Remarks or notes about the occurrence, including substrate information from where the specimen(s) were captured.

## Additional information

### Data analysis

Species richness, frequency and substrate associations were compiled for each locality and elevation band. Species were classified according to their biogeographical origin (native, endemic, introduced) (Gabriel et al. 2010, ABP 2025) and, whenever possible, by their extinction risk following the IUCN categories (Hodgetts et al. 2019).

### Overview of bryophyte and lichen diversity

The opportunistic survey recorded 568 occurrence records on São Jorge Island, comprising 111 cryptogam species, including 102 bryophytes (55 mosses, 44 liverworts and three hornworts) and nine lichens, representing 55 families and 79 genera (Tables 2, 3). Most specimens were identified to species level. *Bryophyta* accounted for the highest species richness, whereas *Marchantiophyta* yielded the largest number of records.

Eight species are newly documented for São Jorge Island, including three bryophytes (*Fissidens dubius*, *Frullania acicularis*, *Phaeoceros laevis*) and five lichens (*Cladonia portentosa*, *Leucodermia leucomelos*, *Pectenia atlantica*, *Pseudocyphellaria aurata*, *Teloschistes flavicans*).

Amongst bryophytes (Table 2), the most frequently recorded liverworts were *Frullania acicularis* and *Heteroscyphus denticulatus*, while *Fissidens asplenioides* and *Hypnum uncinulatum* were the most abundant mosses.

Considering lichens (Table 3), most specimens exhibited a foliose thallus, with fewer showing fruticose forms and some presenting dimorphic characteristics, as observed in *Cladonia* species. Overall, eight genera were identified and the species *Parmotrema reticulatum* and *Teloschistes flavicans* were represented by two specimens each.

### Distribution across elevational zones

**Figure 2.**
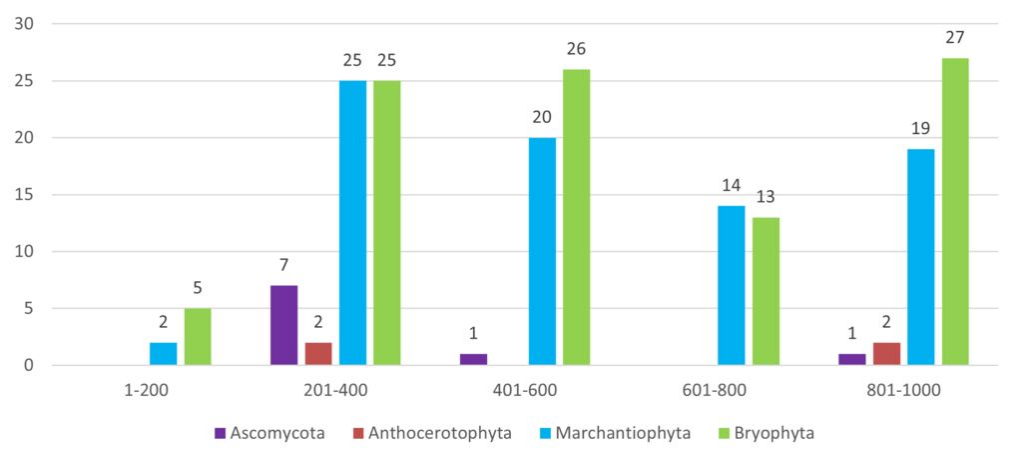
Distribution of species (n = 111) across elevation bands. Number of species of the four taxonomic groups (Ascomycota, *Anthocerotophyta*, *Marchantiophyta* and *Bryophyta*) recorded across five elevation bands on São Jorge Island (Azores).

**Figure 3.**
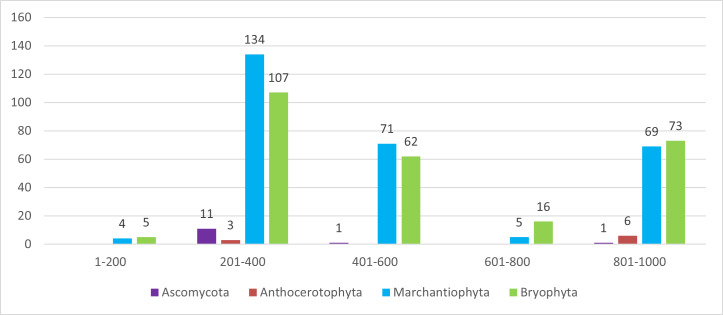
Distribution of specimens (n = 568) across elevation bands. Number of specimens of the four taxonomic groups (*Ascomycota*, *Anthocerotophyta*, *Marchantiophyta* and *Bryophyta*) recorded across five elevation bands on São Jorge Island (Azores).

Native laurel forest supported the highest bryophyte diversity, whereas lichen richness peaked in open, wind-exposed habitats. Species turnover was pronounced along the altitudinal gradient, as observed in other Azores islands (e.g. Gabriel et al. (2025)). In terms of altitude (Figs 2, 3), lichens were collected at lower ranges (200–400 m). In comparison, bryophytes were collected mostly above 300 m, which agrees with previous studies, where bryophytes and lichens have contrasting distributions (Boch et al. 2019).

### Diversity across substrate types

**Figure 4.**
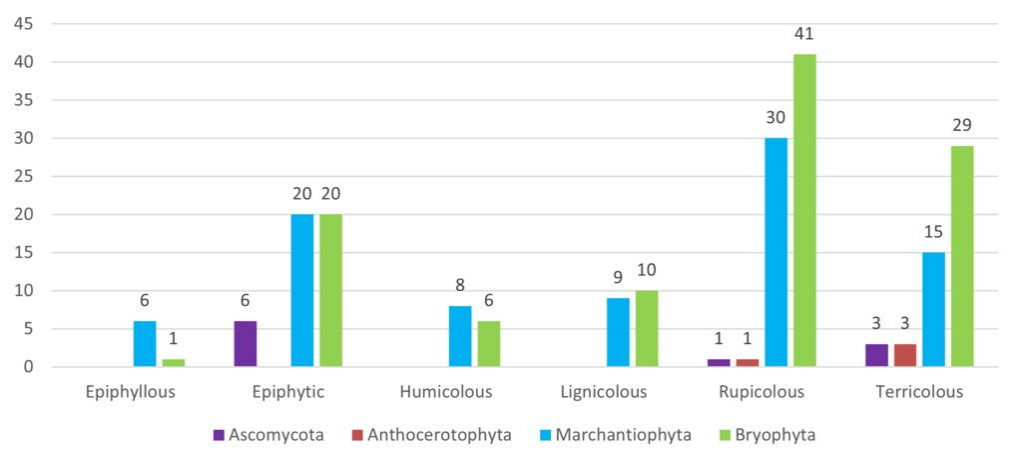
Distribution of species (n = 111) across substrate types. Number of species of the four taxonomic groups (*Ascomycota*, *Anthocerotophyta*, *Marchantiophyta* and *Bryophyta*) recorded on six substrate types on São Jorge Island (Azores).

**Figure 5.**
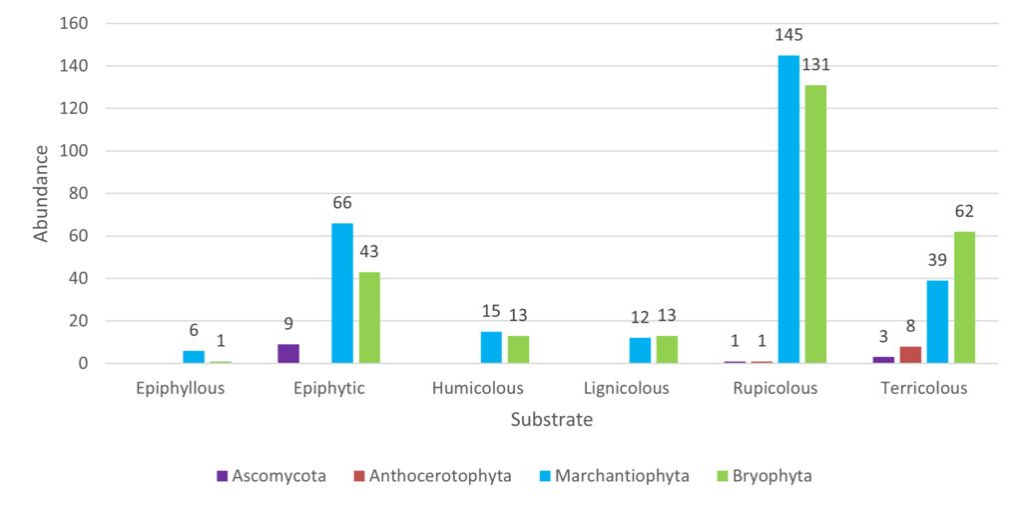
Distribution of specimens (n = 568) across substrate types. Number of specimens of the four taxonomic groups (*Ascomycota*, *Anthocerotophyta*, *Marchantiophyta* and *Bryophyta*) recorded on six substrate types on São Jorge Island (Azores).

With respect to substrate preferences, lichens and liverworts were predominantly collected on tree bark (epiphytes), while mosses were more commonly found on rupicolous and terricolous substrates (Figs 4, 5).

### Biogeographical origin

Within a broader regional context, São Jorge forms part of an archipelago known for its high levels of endemic biodiversity. The São Jorge Natural Park, established in 2011 to protect natural vegetation and preserve unique biodiversity, hosts 188 endemic terrestrial taxa, representing 41% of the Azorean total value of endemic species (GRA 2020). Amongst cryptogams, 10 bryophyte taxa (Gabriel et al. 2010, ABP 2025) and 10 lichens occur exclusively in the Archipelago (Aptroot et al. 2010, Rodrigues et al. 2024). In this study, most recorded taxa are native, comprising three Azorean endemics (*Breutelia azorica*, *Echinodium renauldii*, *Rhynchostegiella azorica*), six Macaronesian endemics and twelve European endemics (Table 2). No invasive bryophyte species were detected during the survey.

### Noteworthy records and habitats

Noteworthy records include *Rhamphidium purpuratum* (Mitten 1870), a European endemic species (GBIF 2025) and *Fontinalis antipyretica*, an aquatic moss reported from only three islands of the Archipelago (Gabriel et al. 2010, ABP 2025). Several conservation-concern species, classified as Vulnerable or Endangered under the IUCN Red List Criteria (Hodgetts et al. 2019), were recorded in São Jorge Island. These include the liverworts *Cololejeunea sintenisii* (EN) and *Frullania microphylla* (VU) and three endangered mosses: *Breutelia azorica*, *Daltonia lindigiana* and *Echinodium renauldii*.

Four conservation concern species, two liverworts (*Frullania microphylla*, *Lejeunea mandonii*) and two mosses (*Andoa berthelotiana*, *Philonotis rigida*), were also found outside of the boundaries of the Island’s Natural Park, occurring at the entrance of a cave (Locality 3). Caves are recognised as one of the most important bryophyte conservation habitats in the Azores (Gabriel et al. 2018, Martín et al. 2019, Cedrés-Perdomo et al. 2024) and the '*Cixius* cave', although located outside the limits of the Natural Park, stands out for its richness in indigenous and threatened species; this place deserves special attention.

Currently, there is no information regarding the IUCN conservation status of lichens.

### Conclusions

This study reinforces the complementary value of opportunistic approaches, which facilitate the collection of rare or inconspicuous taxa, such as hornworts, that may be absent from protocols focused on forest habitats. Although this approach enriches species inventories, enhances local diversity assessments and yields high-quality herbarium material, it may also introduce biases by favouring larger and/or more conspicuous species, both of bryophytes and lichens. Therefore, combining stratified and opportunistic approaches provides the most robust framework for achieving a comprehensive assessment of cryptogam diversity.

## Figures and Tables

**Table 1.** Sampling sites on São Jorge Island (Azores). Columns indicate the site identification code (Local_ID), the presence (✓) or absence (✗) of the site in a Protected Area, name of the locality, elevation (m a.s.l.), latitude and longitude (decimal degrees).

**Local_ID**	**Protected Area**	**Locality**	**Elevation (m a.s.l.)**	**Latitude (N)**	**Longitude (W)**
L01	✓	Rosais, Ponta dos Rosais, zona do Farol.	225.7	38.75338	-28.31188
		Rosais, Ponta dos Rosais, zona do Farol, 0200_P1.	218.6	38.75388	-28.31141
		Rosais, Ponta dos Rosais, zona do Farol, 0200_P2.	217.3	38.75387	-28.31104
L02	✓	Ribeira Seca, Fajã dos Cubres, 0030_P1.	41.2	38.63746	-27.96268
		Ribeira Seca, Fajã dos Cubres.	38.4	38.63737	-27.96230
L03	✗	Norte Grande, Gruta do Cixius ou Gruta das Raízes.	495.2	38.65849	-28.04488
L04	✓	Ribeira Seca, Morro e Pico dos Frades, 0800_P1.	805	38.59037	-27.89559
		Ribeira Seca, Morro e Pico dos Frades, 0800_P2.	805	38.59037	-27.89559
L05	✗	Ribeira Seca, trilho de acesso ao Pico dos Frades.	705.9	38.58494	-27.89884
L06	✗	Santo Antão, Nossa Senhora do Rosário, em talude de caminho.	377.2	38.57320	-27.81194
L07	✓	Topo, Lameiros, 0600_P1.	650	38.57613	-27.84490
		Topo, Lameiros, 0600_P2.	650	38.57608	-27.84496
L08	✗	Santo Antão (São Tomé).	586.5	38.57037	-27.83853
L09	✓	Norte Pequeno, Trilho Pedestre Ladeira das Faias, Estação A.	316.7	38.66099	-28.01286
		Norte Pequeno, Trilho Pedestre Ladeira das Faias, Estação B.	306.6	38.66126	-28.01329
		Norte Pequeno, Trilho Pedestre Ladeira das Faias, Estação C.	303.2	38.66122	-28.01309
		Norte Pequeno, Trilho Pedestre Ladeira das Faias, Estação D.	235.2	38.66176	-28.01298
		Norte Pequeno, Trilho Pedestre Ladeira das Faias, Estação E.	292	38.66164	-28.01386
		Norte Pequeno, Trilho Pedestre Ladeira das Faias, Estação F.	267.3	38.66168	-28.01344
		Norte Pequeno, Trilho Pedestre Ladeira das Faias, Estação G.	314.8	38.66119	-28.01333
		Norte Pequeno, Trilho Pedestre Ladeira das Faias, Estação H.	320.8	38.66075	-28.01251
		Norte Pequeno, Trilho Pedestre Ladeira das Faias, Estação I.	340.1	38.66013	-28.01162
L10	✓	Norte Grande, Caminho para o Pico da Esperança, estação A.	971.3	38.64899	-28.07326
		Norte Grande, Caminho para o Pico da Esperança, estação B.	975.3	38.64910	-28.07321
		Norte Grande, Caminho para o Pico da Esperança, estação C.	975.8	38.64922	-28.07302

**Table 2.** Bryophytes species and subspecies recorded on São Jorge Island, organised by colonisation status and IUCN Red List Category (EN-Endangered; VU-Vulnerable; NT-Near Threatened; LC-Least Concern; DD-Data Deficient). Species marked with * are new records for the Island.

**Colonisation Status**	**Scientific Name**	**IUCN Status**
**Phylum: *Bryophyta***		
Azorean endemic	*Breutelia azorica* (Mitt.) Cardot	EN
	*Echinodium renauldii* (Cardot) Broth.	EN
	*Rhynchostegiella azorica* Hedenäs & Vanderp.	NT
Macaronesian endemic	*Andoa berthelotiana* (Mont.) Ochyra	VU
	*Exsertotheca intermedia* (Brid.) S.Olsson, Enroth & D.Quandt	VU
	*Isothecium prolixum* (Mitt.) M.Stech, Sim-Sim, Tangney & D.Quandt	VU
	*Tetrastichium virens* (Cardot) S.P.Churchill	NT
European endemic	*Hypnum uncinulatum* Jur.	LC
	*Ptychomitrium nigrescens* (Kunze) Wijk & Margad.	LC
	*Rhamphidium purpuratum* Mitt.	NT
	*Tetrastichium fontanum* (Mitt.) Cardot	VU
Native	*Amblystegium serpens* (Hedw.) Schimp. *	LC
	*Atrichum undulatum* (Hedw.) P.Beauv.	LC
	*Brachythecium mildeanum* (Schimp.) Schimp.	LC
	*Brachythecium rutabulum* (Hedw.) Schimp.	LC
	*Campylopus flexuosus* (Hedw.) Brid.	LC
	*Campylopus fragilis* (Brid.) Bruch & Schimp.	LC
	*Campylopus pyriformis* (Schultz) Brid.	LC
	*Campylopus shawii* Wilson	LC
	*Ceratodon purpureus* (Hedw.) Brid.	LC
	*Cyclodictyon laetevirens* (Hook. & Taylor) Mitt.	LC
	*Daltonia lindigiana* Hampe	EN
	*Dicranella howei* Renauld & Cardot	LC
	*Dicranella subulata* (Hedw.) Schimp.	LC
	*Dicranum scottianum* Turner	LC
	*Entosthodon attenuatus* (Dicks.) Bryhn	LC
	*Epipterygium tozeri* (Grev.) Lindb.	LC
	*Fissidens asplenioides* Hedw.	LC
	*Fissidens bryoides* Hedw.	LC
	*Fissidens dubius* P.Beauv. *	LC
	*Fissidens serrulatus* Brid.	LC
	*Fissidens taxifolius* Hedw.	LC
	*Fontinalis antipyretica* Hedw.	LC
	*Heterocladium wulfsbergii* I.Hagen *	LC
	*Hypnum cupressiforme* Hedw.	LC
	*Hypnum jutlandicum* Holmen & E.Warncke	LC
	*Kindbergia praelonga* (Hedw.) Ochyra	LC
	*Leucobryum albidum* (P.Beauv.) Lindb.	DD
	*Leucobryum glaucum* (Hedw.) Ångstr.	LC
	*Myurium hochstetteri* (Schimp.) Kindb.	LC
	*Oxyrrhynchium speciosum* (Brid.) Warnst. *	LC
	*Philonotis fontana* (Hedw.) Brid.	LC
	*Philonotis rigida* Brid.	VU
	*Pleurozium schreberi* (Willd. ex Brid.) Mitt.	LC
	*Polytrichum commune* Hedw.	LC
	*Polytrichum formosum* Hedw.	LC
	*Pseudoscleropodium purum* (Hedw.) M.Fleisch.	LC
	*Ptychostomum pseudotriquetrum* (Hedw.) J.R.Spence & H.P.Ramsay ex Holyoak & N.Pedersen	LC
	*Racomitrium aquaticum* (Brid. ex Schrad.) Brid.	LC
	*Sphagnum auriculatum* Schimp.	LC
	*Sphagnum palustre* L.	LC
	*Sphagnum subnitens* Russow & Warnst.	LC
	*Thamnobryum maderense* (Kindb.) Hedenäs *	NT
	*Thuidium tamariscinum* (Hedw.) Schimp.	LC
	*Tortella nitida* (Lindb.) Broth.	LC
**Phylum: *Marchantiophyta***		
Macaronesian endemic	*Heteroscyphus denticulatus* (Mitt.) Schiffn.	NT
	*Radula wichurae* Steph.	NT
European endemic	*Frullania microphylla* (Gottsche) Pearson	VU
	*Frullania teneriffae* (F.Weber) Nees	LC
	*Marchesinia mackaii* (Hook.) Gray	LC
	*Porella canariensis* (F.Weber) Underw.	LC
	*Radula aquilegia* (Hook.f. & Taylor) Gottsche, Lindenb. & Nees	LC
	*Radula carringtonii* J.B.Jack	NT
	*Radula holtii* Spruce	NT
	*Saccogyna viticulosa* (L.) Dumort.	LC
Native	*Calypogeia arguta* Nees & Mont.	LC
	*Calypogeia fissa* (L.) Raddi	LC
	*Cololejeunea sintenisii* (Steph.) Pócs	EN
	*Colura calyptrifolia* (Hook.) Dumort.	LC
	*Conocephalum conicum* (L.) Dumort.	LC
	*Conocephalum salebrosum* Szweyk., Buczk. & Odrzyk.	LC
	*Diplophyllum albicans* (L.) Dumort.	LC
	*Drepanolejeunea hamatifolia* (Hook.) Schiffn.	LC
	Dumortiera hirsuta (Sw.) Nees subsp. hirsuta	NT
	*Frullania acicularis* Hentschel & von Konrat *	NT
	*Frullania tamarisci* (L.) Dumort.	LC
	*Fuscocephaloziopsis crassifolia* (Lindenb. & Gottsche) Váňa & L.Söderstr.	LC
	*Fuscocephaloziopsis lunulifolia* (Dumort.) Váňa & L.Söderstr.	LC
	Jubula hutchinsiae subsp. javanica (Steph.) Verd.	LC
	*Lejeunea eckloniana* Lindenb.	LC
	*Lejeunea lamacerina* (Steph.) Schiffn.	LC
	*Lejeunea mandonii* (Steph.) Müll.Frib.	VU
	*Lejeunea patens* Lindb.	LC
	*Lophocolea bidentata* (L.) Dumort.	LC
	*Lophocolea fragrans* (Moris & De Not.) Gottsche, Lindenb. & Nees	LC
	*Marchantia paleacea* Bertol.	VU
	*Marsupella sphacelata* (Giesecke ex Lindenb.) Dumort.	LC
	*Metzgeria furcata* (L.) Corda	LC
	*Myriocoleopsis minutissima* (Sm.) R.L.Zhu, Y.Yu & Pócs	LC
	*Nardia scalaris* Gray	LC
	*Odontoschisma sphagni* (Dicks.) Dumort.	LC
	*Pellia epiphylla* (L.) Corda	LC
	*Plagiochila bifaria* (Sw.) Lindenb.	LC
	*Reboulia hemisphaerica* (L.) Raddi	LC
	*Riccardia chamedryfolia* (With.) Grolle	LC
	*Scapania gracilis* Lindb.	LC
	*Scapania nemorea* (L.) Grolle	LC
	*Scapania undulata* (L.) Dumort.	LC
	*Telaranea europaea* J.J.Engel & G.L.Merr.	LC
**Phylum: *Anthocerotophyta***		
Native	*Anthoceros caucasicus* Steph.	LC
	*Anthoceros punctatus* L.	LC
	*Phaeoceros laevis* (L.) Prosk. *	LC

**Table 3.** Lichens recorded on São Jorge Island. Species marked with * are new records for the Island.

**Class**	**Order**	**Family**	**Scientific Name**
* Lecanoromycetes *	* Caliciales *	* Physciaceae *	*Leucodermia leucomelos* (L.) Kalb *
	* Lecanorales *	* Cladoniaceae *	*Cladonia foliacea* (Huds.)
			*Cladonia portentosa* (Dufour) Coem. *
			*Cladonia parasitica* (Hoffm.) Hoffm.
		* Parmeliaceae *	*Parmotrema reticulatum* (Taylor) M. Choisy
			*Usnea* sp.
	* Peltigerales *	* Lobariaceae *	*Pseudocyphellaria aurata* (Ach.) Vain. *
			*Ricasolia virens* (With.) H.H. Blom & Tønsberg
		* Pannariaceae *	*Pectenia atlantica* (Degel.) P.M. Jørg., L. Lindblom, Wedin & S. Ekman *
	* Teloschistales *	* Teloschistaceae *	*Teloschistes flavicans* (Sw.) Norman *

## References

[B13620627] ABP Azorean Biodiversity Portal. https://azoresbioportal.uac.pt/pt/.

[B13624792] Aptroot A., Rodrigues AF., Schumm F., Câmara S., Gabriel R., Borges Paulo A V, Costa A., Cunha R., Gabriel R. (2010). A list of the terrestrial and marine biota from the Azores.

[B13619159] Atherton Ian, Bosanquet Sam, Lawley Mark (2010). Mosses and liverworts of Britain and Ireland: A Field Guide.

[B13619723] Azevedo E B (2001). Condicionantes dinâmicas do clima do Arquipélago dos Açores. Elementos para o seu estudo. Açoreana - Boletim da Sociedade Afonso Chaves.

[B13619754] Boch Steffen, Martins Anabela, Ruas Sara, Fontinha Susana, Carvalho Palmira, Reis Fábio, Bergamini Ariel, Sim‐Sim Manuela (2019). Bryophyte and macrolichen diversity show contrasting elevation relationships and are negatively affected by disturbances in laurel forests of Madeira island. Journal of Vegetation Science.

[B13619767] Borges Paulo A V, Azevedo E B, Borba Alfredo ES, Dinis Francisco O., Gabriel Rosalina, Silva Emiliana, Pereira Henrique Miguel, Domingos Tiago, Vicente Luis, Proença Vânia (2009). Ecossistemas e bem-estar humano em Portugal: Avaliação para Portugal do Millennium Ecosystem Assessment.

[B13619839] Borges Paulo A V, Costa Ana, Cunha Regina, Gabriel Rosalina, Gonçalves Vítor, Martins António Frias, Melo Ireneia, Parente Manuela, Raposeiro Pedro, Rodrigues Pedro, Santos Ricardo Serrão, Silva Luís, Vieira Paulo, Vieira Virgílio (2010). A list of the terrestrial and marine biota from the Azores.

[B13750452] Borregaard M. K., Amorim I. R., Borges P. A.V., Cabral J. S., Fernández-Palacios J. M., Field R., Heaney L. R., Kreft H., Matthews T. J., Olesen J. M., Price J., Rigal F., Steinbauer M. J., Triantis K. A., Valente L., Weigelt P., Whittaker R. J. (2017). Oceanic island biogeography through the lens of the general dynamic model: assessment and prospect. Biological Reviews.

[B13619887] Broughton R K, Pocock M J O (2022). The opportunities for semi-structured and effort recording to enhance the value of biological recording by volunteers. https://data.jncc.gov.uk/data/57e64ede-78b2-43dd-8e50-35555874fdbc/tsda-biological-recording-opportunities.pdf.

[B13619913] Casas Creu, Brugués Montserrat, Cros Rosa M, Sérgio Cecília, Infante Marta (2009). Handbook of liverworts and hornworts of the Iberian Peninsula and the Balearic Islands: illustrated keys to genera and species.

[B13619897] Casas Creu, Brugués Montserrat, Cros Rosa M, Sérgio Cecília (2020). Handbook of mosses of the Iberian Peninsula and the Balearic Islands: illustrated keys to genera and species.

[B13619964] Cedrés-Perdomo Ruymán David, Polaíno-Martín Clara, Jennings Laura, Gabriel Rosalina (2024). Seeking a hideout: Caves as refuges for various functional groups of bryophytes from Terceira Island (Azores, Portugal). Diversity.

[B13619998] Coelho Márcia C. M., Gabriel Rosalina, Ah-Peng Claudine (2023). Characterizing and quantifying water content in 14 species of bryophytes present in Azorean native vegetation. Diversity.

[B13620010] DRAAC Caraterização Climática. Relatório do Estado do Ambiente dos Açores (REAA). https://rea.azores.gov.pt/reaa/9/clima-e-alteracoes-climaticas/1482/caraterizacao-climatica.

[B13750277] Elias Rui B., Gil Artur, Silva Luís, Fernández-Palacios José M., Azevedo Eduardo B., Reis Francisco (2016). Natural zonal vegetation of the Azores Islands: characterization and potential distribution. Phytocoenologia.

[B13620027] Ellis Christopher J., Asplund Johan, Benesperi Renato, Branquinho Cristina, Di Nuzzo Luca, Hurtado Pilar, Martínez Isabel, Matos Paula, Nascimbene Juri, Pinho Pedro, Prieto María, Rocha Bernardo, Rodríguez-Arribas Clara, Thüs Holger, Giordani Paolo (2021). Functional traits in lichen ecology: A review of challenge and opportunity. Microorganisms.

[B13624542] Forjaz Victor Hugo, Azevedo Eduardo, Afonso Pedro, Borges Paulo Jorge (2004). Atlas básico dos Açores.

[B13620047] Gabriel R., Bates J. W. (2005). Bryophyte community composition and habitat specificity in the natural forests of Terceira, Azores. Plant Ecology.

[B13624696] Gabriel Rosalina, Sjögren E., Schumacker R., Borges Paulo A V, Costa A., Cunha R., Gabriel R. (2010). A list of the terrestrial and marine biota from the Azores.

[B13620519] Gabriel Rosalina, Sim-Sim Maria Manuela, González-Mancebo Juana María (2018). Conservation concern’ bryophytes find refuge on cave entrances in the Azores. ARPHA Conference Abstracts.

[B13620056] Gabriel Rosalina, Pimentel César, Claro David, Brito Mariana, Díaz-Castillo Javier, Sérgio Cecília, Sim-Sim Manuela, Borges Paulo (2019). Biota of coastal wetlands of Praia da Vitória (Terceira Island, Azores): Part 2 - Bryophytes. Biodiversity Data Journal.

[B13620085] Gabriel Rosalina, Morgado Leila, Henriques Débora, Coelho Márcia, Hernández-Hernández Raquel, Borges Paulo (2024). The MOVECLIM – AZORES project: Bryophytes from Terceira Island along an elevation gradient. Biodiversity Data Journal.

[B13620069] Gabriel Rosalina, Morgado Leila, Borges Paulo, Coelho Márcia, Aranda Silvia, Henriques Débora, Sérgio Cecília (2024). The MOVECLIM – AZORES project: Bryophytes from Pico Island along an elevation gradient. Biodiversity Data Journal.

[B13620719] Gabriel Rosalina, Silveira Gabriela (2025). The MOVECLIM – AZORES project: Ad hoc bryophyte and lichen collections from São Jorge Island (2014).

[B13830015] Gabriel Rosalina, Morgado Leila Nunes, Poponessi Silvia, Henriques Débora S. G., Coelho Márcia C. M., Silveira Gabriela M., Borges Paulo A. V. (2025). Diversity and distribution of bryophytes along an altitudinal gradient on Flores Island (Azores, Portugal). Plants.

[B13624221] GBIF *Rhamphidium purpuratum* (Mitt.). https://www.gbif.org/species/5282419.

[B13750443] Gillespie R. G., Claridge E. M., Roderick G. K. (2008). Biodiversity dynamics in isolated island communities: interaction between natural and human-mediated processes. Molecular Ecology.

[B13750613] González-Mancebo Juana M., Gabriel Rosalina, Otto Rüdiger, Sim-Sim Manuela, Luís Leena Margarida, Sérgio Cecília, Caujapé-Castells J, Nieto Feliner G, Fernández Palacios JM (2013). A comparison of bryophyte diversity in the Macaronesian Islands: island versus habitat approach. Proceedings of the Amurga international conferences on island biodiversity 2011.

[B13620445] GRA São Jorge Nature Park. https://parquesnaturais.azores.gov.pt/en/parques/6.

[B13620208] Guerra Juan, Cano Maria J, Brugués Montserrat (2018). Flora Briofítica Ibérica.

[B13624385] Hodgetts Nick, Cálix Marta, Englefield Eve, Fettes Nicholas, Criado Mariana García, Patin Lea (2019). A miniature world in decline European Red List of: Mosses, liverworts and hornworts.

[B13750518] MacArthur Robert H, Wilson Edward O (1967). The theory of island biogeography.

[B13620487] Martín Clara Polaino, Gabriel Rosalina, Jennings Laura, Amorim Isabel R, Henar-Sánchez Marina, Prieto Alejandra Ros, Peroni Cristina, Dias Eduardo, Borges Paulo A V, Pereira Fernando (2019). Crescendo na obscuridade: briófitos da Ilha Terceira em ambientes cavernícolas. Pingo de Lava.

[B13737918] Mitten W., Godman F. (1870). Natural history of the Azores on Western Islands.

[B13829998] Mouton L., Patiño J., Carine M., Rumsey F., Sequeira M. M., González-Mancebo J. M., Gabriel R. M.A., Hardy O. J., Sim-Sim M., Reyes-Betancort J. A., Collart F., Vanderpoorten A. (2023). Patterns and drivers of beta diversity across geographic scales and lineages in the Macaronesian flora. Journal of Biogeography.

[B13620427] Oksanen Ilona (2006). Ecological and biotechnological aspects of lichens. Applied Microbiology and Biotechnology.

[B13620436] Pakeman Robin J., Brooker Rob W., O'Brien David, Genney David (2019). Using species records and ecological attributes of bryophytes to develop an ecosystem health indicator. Ecological Indicators.

[B13620462] Paton Jean A. (1999). The liverwort flora of the British Isles.

[B13620502] Rodrigues António Félix, Videira Sandra, Aptroot André, Gabriel Rosalina (2024). Lichen novelties from Corvo Island (Azores, Portugal). Biodiversity Data Journal.

[B13620511] Schumacker René, Váňa Jiří (2005). Identification keys to the liverworts and hornworts of Europe and Macaronesia (distribution and status).

[B13620537] Smith A J E (2004). The moss flora of Britain and Ireland.

